# Social adaptability and its associations with eHealth literacy and physical activity among first- and second-year medical students in China: a cross-sectional study

**DOI:** 10.3389/fpubh.2026.1807832

**Published:** 2026-08-24

**Authors:** Mengying Wu, Yupeng Jiang, Le Wang, Shouzhi Wu, Jun Sun, Zhixuan Tao, Zihao Wang, Sihang Xun, Yanan Zhang, Ergang Zhu, Ningfang Zhu, Guangjian Li, Xugui Sun

**Affiliations:** 1 Pharmacy Department, The Second People's Hospital, Wuhu, Wuhu, Anhui, China; 2 Second Clinical Medical College, Xuzhou Medical University, Xuzhou, Jiangsu, China; 3 School of Nursing, Wannan Medical College, Wuhu, Anhui, China; 4 Physical Education Teaching and Research Office, Wannan Medical College, Wuhu, Anhui, China; 5 Hospital Infection Control Department, Changzhou Dean Hospital, Changzhou, Jiangsu, China; 6 Graduate School, Wannan Medical College, Wuhu, Anhui, China; 7 Psychology Department, Changzhou Dean Hospital, Changzhou, Jiangsu, China

**Keywords:** cross-sectional study, eHealth literacy, first- and second-year students, physical activity, social adaptability

## Abstract

**Background:**

First- and second-year medical students face multiple challenges during the transition to university, including increased academic demands, changes in learning environments, and difficulties in social adaptation. Social adaptability is closely related to students' mental health and psychosocial functioning. eHealth literacy and physical activity have been identified as important behavioral and cognitive factors that may be associated with social adaptability.

**Objective:**

This study aimed to examine the level of social adaptability among first- and second-year medical students and to explore its associations with eHealth literacy and physical activity.

**Methods:**

An online questionnaire survey was conducted in two waves, in October 2023 and 2024, using a stratified online convenience sampling approach among first- and second-year students at Wannan Medical College in Wuhu, Anhui, China. The Social Adaptation Inventory, eHealth Literacy Scale, and Physical Activity Rating Scale-3 were used to assess students' social adaptability, eHealth literacy, and physical activity.

**Results:**

Among the 2,026 participants, social adaptability was positively correlated with physical activity (Spearman's *r*_s_ = 0.219, *P* < 0.001) and eHealth literacy (Spearman's *r*_s_ = 0.348, *P* < 0.001). In the final hierarchical regression model, physical activity (β = 0.124, *P* < 0.001) and eHealth literacy (β = 0.294, *P* < 0.001) remained positively associated with social adaptability after adjustment for gender, year of study, place of origin, program duration, student leadership status, and survey wave. The final model explained 16.9% of the variance in social adaptability (*R*^2^ = 0.169; adjusted *R*^2^= 0.166).

**Conclusion:**

Physical activity and eHealth literacy were independently associated with social adaptability among first- and second-year medical students. However, given the cross-sectional design and the modest explanatory capacity of the model, these findings should be interpreted as associations and require confirmation in longitudinal and intervention studies.

## 1 Introduction

The COVID-19 pandemic disrupted higher education, altered learning environments, and reduced opportunities for face-to-face interaction among university students. Previous studies reported that these disruptions were associated with psychological distress, social isolation, learning difficulties, and changes in students' academic and interpersonal experiences (1, 2). However, the extent to which these effects persisted after the resumption of regular campus life may have varied across individuals and student cohorts. Therefore, in the present study, the pandemic is considered part of the broader educational context rather than a directly measured explanation for students' current social adaptability.

Social adaptability refers to an individual's capacity to adjust behaviors, emotions, and interpersonal interactions in response to environmental demands, thereby achieving a dynamic balance between personal needs and social expectations (3). In the context of university life, social adaptability encompasses multiple domains, including interpersonal relationships, academic adjustment, self-management, and psychological regulation. It represents an important aspect of students' psychosocial functioning and their capacity to respond to academic and interpersonal demands in university settings (4, 5).

The transition from high school to university represents an important developmental period during which students must adapt to new academic expectations, increased independence, changing social relationships, and unfamiliar institutional environments (6). Research involving first-year health professions students has identified challenges in academic, social, and personal-emotional adjustment during the transition to university (7). Similarly, research among first-year medical students has characterized college adjustment as a multidimensional process involving academic, social, and personal-emotional adaptation (8). These challenges may be particularly relevant to first- and second-year medical students, who must simultaneously manage intensive coursework, professional expectations, peer relationships, and independent living. Identifying potentially modifiable factors associated with social adaptability may therefore provide useful evidence for supporting students' psychosocial adjustment.

eHealth literacy refers to an individual's ability to seek, find, understand, evaluate, and apply health information obtained from electronic sources to address health-related problems (9). This capacity may be relevant to university students' health-related decision-making and self-management because electronic sources have become an important channel through which students obtain health information. Physical activity may also be relevant to psychosocial functioning. A recent systematic review identified social support, social connection, self-esteem, self-efficacy, and resilience as potential mechanisms linking physical activity with mental health and wellbeing (10). However, the associations of eHealth literacy and physical activity with social adaptability among early-year medical students remain insufficiently understood. Accordingly, the present study aimed to examine the associations of physical activity and eHealth literacy with social adaptability among first- and second-year medical students and to determine whether these associations remained significant after adjustment for selected sociodemographic characteristics and survey wave.

## 2 Methods

### 2.1 Study design and participants

This study adopted a cross-sectional survey design. Participants were first- and second-year undergraduate students enrolled in medical programs at Wannan Medical College, China. Data were collected through two concentrated online survey waves, one in October 2023 and the other in October 2024. A stratified online convenience sampling approach with voluntary participation was used. Students were stratified according to medical program and year of study, and the questionnaire link was distributed within each program–year stratum at the beginning of the autumn semester. Both survey waves included first- and second-year students, and participants completed the questionnaire independently.

The survey was administered anonymously through an online questionnaire platform. No personally identifying information, such as names or student identification numbers, was collected. Participants completed the questionnaire using their own electronic devices outside regular class time, and neither teachers nor investigators were present during questionnaire completion. Both survey waves were conducted at the beginning of the autumn semester and avoided examination periods and major holidays.

A total of 2,100 questionnaires were submitted. No questionnaires were excluded because of missing data. Questionnaires completed in less than 100 s were considered invalid because this duration was deemed insufficient for participants to read and respond carefully to all questionnaire items. Based on this data-quality criterion, 74 questionnaires were excluded, leaving 2,026 valid questionnaires for the final analysis and yielding a valid questionnaire rate of 96.5%. Of the included participants, 955 (47.1%) completed the survey in October 2023 and 1,071 (52.9%) completed it in October 2024. Because both surveys were conducted in the same calendar month and at a comparable stage of the academic year, potential variation related to season, examination periods, and major holidays was reduced.

Inclusion criteria were as follows: (1) enrollment as a first- or second-year medical student; (2) ability to understand and complete the questionnaire independently; and (3) voluntary participation with informed consent. Exclusion criteria included self-reported history of severe mental disorders, intellectual disability, or serious physical illness that could interfere with survey completion.

The study protocol was reviewed and approved by the Ethics Review Committee of Wannan Medical College [Approval No.: (2023) 206]. Electronic informed consent was obtained from all adult participants before completion of the online questionnaire. Among the 2,026 included participants, 24 students (1.2%) were younger than 18 years, and all were 17 years old. For these participants, informed consent was obtained from a parent or legal guardian, together with assent from the participant.

### 2.2 Survey instruments

#### 2.2.1 General information questionnaire

A self-designed general information questionnaire was used to collect demographic data from the participants, including gender, age, year of study, place of origin, academic system, student leadership roles, and whether they are only children.

#### 2.2.2 Social Adaptability Inventory (SAI)

The Social Adaptability Inventory (SAI), developed by Zheng Richang, consists of 20 items encompassing five dimensions: peer relationship skills, self-management skills, learning skills, obedience skills, and willingness expression skills. Items are rated on a three-point scale (“Yes,” “Uncertain,” and “No”), scored as −2, 0, and 2, respectively (11). Based on the total score, social adaptability is classified into five levels: very poor ( ≤ 5), poor (6–16), average (17–28), good (29–34), and excellent (35–40) (12). The SAI has been widely used in Chinese populations to assess social adaptability. In a Chinese sample, the Cronbach's α coefficient of the SAI was 0.787 (13).

#### 2.2.3 Physical Activity Rating Scale-3 (PARS-3)

The Physical Activity Rating Scale-3 (PARS-3), originally developed by Koyo Hashimoto in Japan and subsequently revised by Liang Deqing for use in China, is designed to assess individuals' physical activity levels in terms of intensity, duration, and frequency. The total physical activity score is calculated using the formula: Physical Activity Level = Exercise Intensity × (Exercise Time-1) × Exercise Frequency. Each component is rated on a five-point scale (1–5), yielding a total score ranging from 0 to 100, with higher scores indicating higher levels of physical activity participation (14). The PARS-3 has been widely used in Chinese populations, and in a Chinese sample, the Cronbach's α coefficient was 0.80 (15). This study used PARS-3 to assess the physical activity status of university students.

#### 2.2.4 eHealth literacy scale (eHeals)

The eHealth Literacy Scale (eHEALS), developed by Norman et al. (16) and subsequently adapted by Guo Shuaijun for use in China, consists of eight items covering three dimensions: the ability to apply online health information and services, the ability to evaluate such information, and the ability to make health-related decisions based on it. Items are rated on a five-point Likert scale ranging from 1 (“strongly disagree”) to 5 (“strongly agree”), with higher scores indicating higher levels of eHealth literacy. The Chinese version of the eHEALS has been widely used in Chinese populations, and in a Chinese sample, the Cronbach's α coefficient was 0.913 (17). This study used the eHEALS to assess participants' self-reported eHealth literacy.

### 2.3 Statistical analysis

Data analysis was performed using SPSS 22.0 software. Categorical variables were expressed as frequencies and percentages, and chi-square (χ^2^) tests were used for group comparisons. After normality testing, normally distributed continuous variables were presented as mean ± standard deviation (x ± s), and independent-sample *t*-tests were applied for comparisons between two groups. Non-normally distributed variables were described using medians and interquartile ranges (P25, P75), and the Mann–Whitney *U*-test was used for between-group comparisons. Spearman correlation analysis was conducted to examine associations among variables. Hierarchical multiple linear regression analysis was conducted with social adaptability as the dependent variable. Model 1 included gender, year of study, place of origin, program duration, student leadership status, and survey wave. Physical activity was added in Model 2, and eHealth literacy was further added in Model 3. A two-tailed *p*-value of < 0.05 was considered statistically significant.

## 3 Results

### 3.1 General information of first- and second-year medical students

A total of 2,026 participants were included in this study, with ages ranging from 17 to 23 years, and a mean age of 19.37 ± 0.89 years. Among the participants, 779 were male (38.5%) and 1,247 were female (61.5%). There were 1,355 first-year students (66.9%) and 671 second-year students (33.1%). In terms of residency, 862 were from urban areas (42.5%) and 1,164 from rural areas (57.5%). Regarding the academic system, 938 students were enrolled in a 4-years program (46.3%), and 1,088 students in a 5-years program (53.7%). Of the participants, 595 were student leaders (29.4%) and 1,431 were non-leaders (70.6%). Additionally, 542 students were only children (26.8%), while 1,484 were not (73.2%). Regarding survey timing, 955 participants (47.1%) completed the survey in October 2023, whereas 1,071 participants (52.9%) completed the survey in October 2024. Both survey waves included first- and second-year students see Table 1 for details.

**Table 1:** Comparison of social adaptability, physical activity, and eHealth literacy scores among first- and second-year medical students [M (P_25_, P_75_)].

Project	Categorization	*n* (%)	Social adaptability	Physical activity	eHealth literacy
Gender					
	Male	779(38.5)	2 (0, 12)	18 (8, 36)	31 (26, 32)
	Female	1247(61.5)	2 (−4, 10)	9 (4,18)	29 (26, 32)
	*Z*		−4.754	−12.736	−2.973
	*P*		< 0.001	< 0.001	0.003
Year of study					
	First-year students	1355(66.9)	2 (−2, 12)	12 (6,24)	30 (26, 32)
	Second-year students	671(33.1)	0 (−4, 8)	12 (6,24)	31 (26, 32)
	*Z*		−2.252	−0.221	−2.029
	*P*		0.024	0.825	0.042
Place of origin					
	Urban	862(42.5)	2 (0, 12)	12 (6,24)	31 (27, 32)
	Rural	1164(57.5)	0 (−4, 8)	12 (6,24)	29 (25, 32)
	*Z*		−5.072	−1.197	−6.184
	*P*		< 0.001	0.231	< 0.001
Program duration					
	4-year	938(46.3)	2 (−4, 10)	12 (6,24)	29 (26, 32)
	5-year	1088(53.7)	2 (−2, 12)	12 (6,24)	30 (26, 32)
	*Z*		−4.229	−0.009	−2.264
	*P*		< 0.001	0.993	0.024
Student leadership status					
	Yes	595(29.4)	4 (0, 16)	12 (6, 32)	31 (26, 32)
	No	1431(70.6)	0 (−4, 8)	10 (6,24)	30 (25, 32)
	*Z*		−8.003	−4.889	−4.007
	*P*		< 0.001	< 0.001	< 0.001
Only-child status					
	Yes	542(26.8)	2 (−2, 10)	12 (6,27)	31 (26, 32)
	No	1484(73.2)	2 (−2, 10)	11 (5.25, 24)	30 (26, 32)
	*Z*		−0.712	−3.801	−2.737
	*P*		0.476	< 0.001	0.006
Survey wave					
	October 2023	955 (47.1)	2 (−2, 12)	12 (4,24)	30 (25, 32)
	October 2024	1,071 (52.9)	2 (−2, 10)	12 (6,24)	30 (26, 32)
	*Z*		−1.241	−1.735	−0.112
	*P*		0.215	0.083	0.911

### 3.2 Comparison of social adaptability, physical activity, and eHealth literacy scores among first- and second-year medical students

According to the predefined SAI score ranges, 1,254 participants (61.9%) scored in the very poor range and 476 (23.5%) in the poor range. A further 223 participants (11.0%), 44 (2.2%), and 29 (1.4%) scored in the average, good, and excellent ranges, respectively. Differences in social adaptability, physical activity, and eHealth literacy scores across participant characteristics are presented in Table 1. Social adaptability scores were significantly higher among male students, first-year students, students from urban areas, those enrolled in 5-years programs, and student leaders (all *P* < 0.05). Physical activity scores were significantly higher among male students, student leaders, and only children than among their respective counterparts (all *P* < 0.05). eHealth literacy scores were significantly higher among male students, second-year students, students from urban areas, those enrolled in 5-years programs, student leaders, and only children (all *P* < 0.05). No statistically significant differences were observed between the October 2023 and 2024 survey waves in social adaptability, physical activity, or eHealth literacy scores (all *P* > 0.05) see Table 1 for details.

### 3.3 Correlation analysis of social adaptability, physical activity, and ehealth literacy among first- and second-year medical students

In this study, social adaptability among first- and second-year medical students was positively correlated with both eHealth literacy and physical activity scores (both *P* < 0.05). Additionally, eHealth literacy scores were positively correlated with physical activity scores (*P* < 0.05) see Table 2 for details.

**Table 2:** Spearman correlations among social adaptability, physical activity, and eHealth literacy in first- and second-year medical students (r_s_).

Variable	$\bar{\text{x}}$ ±s	Social adaptability	Physical activity	eHealth literacy
Social adaptability	3.96 ± 11.80	1		
Physical activity	17.83 ± 18.92	0.219^**^	1	
eHealth literacy	29.28 ± 5.00	0.348^**^	0.201^**^	1

^*^P < 0.05, ^**^P < 0.01.

### 3.4 Hierarchical multiple linear regression analysis of factors associated with social adaptability

Hierarchical multiple linear regression analysis was conducted with social adaptability as the dependent variable. Gender, year of study, place of origin, program duration, student leadership status, and survey wave were entered in Model 1. Physical activity was added in Model 2, and eHealth literacy was further added in Model 3. The coding scheme for the variables included in the regression analysis is presented in Table 3.

**Table 3:** Variable coding scheme.

Variable	Coding scheme
Gender	1 = Male, 2 = Female
Year of study	1 = Freshman, 2 = Sophomore
Place of origin	1 = Urban, 2 = Rural
Program duration	1 = 4-year, 2 = 5-year
Student leadership status	1 = Yes, 2 = No
Survey wave	1 = October 2023
	2 = October 2024
Physical activity	Original value
eHealth literacy	Original value

The overall regression models were statistically significant. Model 1, which included sociodemographic characteristics and survey wave, explained 6.0% of the variance in social adaptability (*R*^2^ = 0.060, adjusted *R*^2^ = 0.057, *F* = 21.315, *P* < 0.001). After physical activity was added in Model 2, the explained variance increased to 8.7% (*R*^2^ = 0.087, adjusted *R*^2^ = 0.084, *F* = 27.626, *P* < 0.001). With the addition of eHealth literacy in Model 3, the explained variance further increased to 16.9% (*R*^2^ = 0.169, adjusted *R*^2^ = 0.166, *F* = 51.304, *P* < 0.001).

In Model 1, gender, year of study, place of origin, program duration, and student leadership status were significantly associated with social adaptability, whereas survey wave was not significantly associated with social adaptability (*B* = 0.834, β = 0.035, *P* = 0.130). In Model 2, physical activity was positively associated with social adaptability after adjustment for sociodemographic characteristics and survey wave (*B* = 0.110, β = 0.176, *P* < 0.001).

In the final model, survey wave remained non-significant (*B* = 0.357, SE = 0.520, β = 0.015, *P* = 0.492). Physical activity (*B* = 0.077, SE = 0.014, β = 0.124, *P* < 0.001) and eHealth literacy (*B* = 0.693, SE = 0.049, β = 0.294, *P* < 0.001) were independently and positively associated with social adaptability after adjustment for sociodemographic characteristics and survey wave. The variance inflation factor values ranged from 1.014 to 1.174, indicating no evidence of multicollinearity among the independent variables. Detailed results are presented in Table 4.

**Table 4:** Hierarchical multiple linear regression analysis of factors associated with social adaptability among first- and second-year medical students.

Variable	*B*	SE	β	*t*	95% CI for B	*P*	*R* ^2^	Adjusted *R*^2^	*F*
Model 1 (Sociodemographic characteristics and survey wave)							0.060	0.057	21.315^**^
Gender	−2.344	0.530	−0.097	−4.426	[−3.382, −1.305]	< 0.001			
Year of study	−1.709	0.545	−0.068	−3.136	[−2.777, −0.640]	0.002			
Place of origin	−2.006	0.523	−0.084	−3.835	[−3.032, −0.980]	< 0.001			
Program duration	1.462	0.527	0.062	2.774	[0.429, 2.496]	0.006			
Student leadership status	−4.704	0.600	−0.182	−7.838	[−5.881, −3.527]	< 0.001			
Survey wave	0.834	0.551	0.035	1.513	[−0.247, 1.916]	0.130			
Constant	13.304	3.071		4.332	[7.281, 19.327]	< 0.001			
Model 2 (Model 1 + Physical Activity)							0.087	0.084	27.626^**^
Gender	−1.108	0.545	−0.046	−2.034	[−2.177, −0.040]	0.042			
Year of study	−1.624	0.537	−0.065	−3.023	[−2.677, −0.570]	0.003			
Place of origin	−1.889	0.516	−0.079	−3.664	[−2.900, −0.878]	< 0.001			
Program duration	1.687	0.520	0.071	3.243	[0.667, 2.707]	0.001			
Student leadership status	−4.116	0.596	−0.159	−6.906	[−5.285, −2.947]	< 0.001			
Survey wave	0.555	0.544	0.023	1.019	[−0.513, 1.623]	0.308			
Physical activity	0.110	0.014	0.176	7.852	[0.082, 0.137]	< 0.001			
Constant	7.463	3.116		2.395	[1.352, 13.574]	0.017			
Model 3 (Model 2 + eHealth Literacy)							0.169	0.166	51.304^**^
Gender	−1.158	0.520	−0.048	−2.226	[−2.178, −0.138]	0.026			
Year of study	−1.742	0.513	−0.069	−3.399	[−2.748, −0.737]	0.001			
Place of origin	−1.113	0.495	−0.047	−2.249	[−2.084,−0.142]	0.025			
Program duration	1.510	0.497	0.064	3.041	[0.536, 2.484]	0.002			
Student leadership status	−3.624	0.570	−0.140	−6.358	[−4.742, −2.506]	< 0.001			
Survey wave	0.357	0.520	0.015	0.687	[−0.662, 1.377]	0.492			
Physical activity	0.077	0.014	0.124	5.715	[0.051, 0.104]	< 0.001			
eHealth literacy	0.693	0.049	0.294	14.077	[0.596, 0.789]	< 0.001			
Constant	−12.965	3.309		−3.917	[−19.455, −6.474]	< 0.001			

^*^P < 0.05, ^**^P < 0.01; *B*, unstandardized regression coefficient; SE, standard error; CI, confidence interval; β, standardized regression coefficient; Model 1 included sociodemographic characteristics and survey wave; Model 2 additionally included physical activity; Model 3 additionally included eHealth literacy.

## 4 Discussion

### 4.1 Social adaptability of first- and second-year medical students

The results of this study indicate that the social adaptability scores of first- and second-year medical students are generally at a relatively low level. In the present sample, the average social adaptability score among the 2,026 medical students was 3.96 ± 11.80. A previous study reported a higher social adaptability score among another university student sample (8.167 ± 13.64) (18). However, direct comparisons should be interpreted cautiously because of differences in participant characteristics, academic disciplines, educational settings, and survey periods. The present findings are generally consistent with previous research on social adaptability among medical students (19).

The relatively low level of social adaptability observed among first- and second-year medical students may be related to multiple contextual factors. One possible but unmeasured contextual factor is the educational and social disruption experienced by some students during the COVID-19 pandemic. During this period, some participants may have experienced online learning, reduced face-to-face interaction, and substantial lifestyle changes during late adolescence. These disruptions may have limited opportunities for campus-based activities and peer interaction, which are important for the development of social skills, emotional regulation, and self-management abilities (2).

Some students may therefore have continued to experience difficulties in interpersonal communication, emotional adjustment, and adaptation to collective environments after regular campus-based teaching resumed. Previous research has indicated that reduced social interaction and increased psychological stress during the pandemic were associated with anxiety and depressive symptoms, which may also be related to challenges in social adaptability (20). However, pandemic-related experiences were not directly assessed in the present study, and no pre-pandemic comparison group was available. Therefore, this interpretation is speculative and should be regarded only as a possible contextual explanation rather than as a conclusion supported by the present findings.

In addition to the broader pandemic-related context, academic demands may also be associated with social adaptability among early-year medical students. Medical education is characterized by intensive coursework, high performance expectations, and early exposure to professional identity formation. Students are required to adjust simultaneously to academic routines, social environments, and increasing independence. Previous studies have suggested that heightened academic stress among medical students is associated with adverse psychological outcomes and difficulties in psychosocial adjustment (21). These academic and developmental demands may collectively be related to challenges in social adaptability during the early stages of medical training.

### 4.2 Factors associated with social adaptability among first- and second-year medical students

Based on correlation and regression analyses, several factors were found to be associated with social adaptability among first- and second-year medical students. These factors included gender, year of study, place of origin, program duration, student leadership status, eHealth literacy, and physical activity.

Gender was associated with social adaptability, with male students reporting higher social adaptability scores than female students. However, the mechanisms underlying this difference cannot be determined from the present data. The observed association may reflect multiple factors, including differences in physical activity participation, social expectations, response patterns to self-report measures, or psychological and interpersonal characteristics not assessed in this study. Although gender remained significantly associated with social adaptability after adjustment for physical activity and other included variables, this finding does not establish that gender itself directly determines social adaptability. Further research incorporating more detailed measures of coping styles, psychological characteristics, social support, and gender-related experiences is needed to clarify this association.

First-year students reported higher social adaptability scores than second-year students, which is consistent with a previous study (22). However, this finding should be interpreted cautiously. Because the study used two separate cross-sectional survey waves rather than following the same students over time, the observed difference cannot be interpreted as evidence that social adaptability declines from the first to the second year. Differences in cohort composition, academic workload, semester-related experiences, or other unmeasured factors may have contributed to the result. Although both survey waves were conducted in October and survey wave was not significantly associated with social adaptability in the adjusted model, residual cohort and timing effects cannot be completely excluded. Longitudinal studies are needed to clarify how social adaptability changes across years of medical education.

Academic system characteristics and student leadership roles were also associated with social adaptability. Students enrolled in 5-year programs, such as clinical medicine, dentistry, and anesthesiology, typically experience sustained academic demands and examination pressure, which may influence their social adjustment processes (23). In contrast, students holding leadership roles may have more opportunities to participate in organizational activities and interpersonal communication. Broader evidence suggests that psychological and interpersonal characteristics are related to social adjustment (24); however, the specific mechanisms underlying the association between student leadership status and social adaptability were not directly assessed in the present study.

eHealth literacy was positively associated with social adaptability among early-year medical students. Students with higher eHealth literacy may be better able to access, evaluate, and apply online health information, which may support informed health-related decision-making and self-management (25). However, because the present study was cross-sectional, the direction of this association cannot be determined. Physical activity was also positively associated with social adaptability. Previous research suggests that physical activity is associated with stress reduction, emotional wellbeing, and opportunities for social interaction, which may partly explain its association with social adaptability (26).

Nevertheless, the explanatory capacity of the final regression model should be interpreted cautiously. The final model accounted for 16.9% of the variance in social adaptability, with an adjusted *R*^2^ of 16.6%, indicating that most of the variability remained unexplained. Therefore, physical activity and eHealth literacy should be regarded as relevant correlates rather than dominant determinants of social adaptability. Other unmeasured factors, such as personality characteristics, baseline mental health, family environment, academic stress, perceived social support, interpersonal relationships, and campus environment, may also contribute to social adaptability.

### 4.3 Practical implications for supporting social adaptability among first- and second-year medical students

The present findings identify physical activity and eHealth literacy as two potentially modifiable factors associated with social adaptability among early-year medical students. Given the modest explanatory capacity of the final model (adjusted *R*^2^ = 16.6%), these factors should be viewed as potentially relevant components of a broader range of factors associated with social adaptability, rather than as primary or sufficient intervention targets. However, because of the cross-sectional design, these findings should not be interpreted as evidence that increasing physical activity or eHealth literacy will necessarily improve social adaptability. Instead, they identify potential areas for future longitudinal and intervention research.

Physical activity may provide opportunities for peer interaction, cooperation, and stress regulation. Previous research has linked physical activity with better mental health and psychological wellbeing among adolescents and university students (26, 27). Universities may therefore consider providing accessible and varied opportunities for recreational and group-based physical activity. Nevertheless, the present study did not compare different types of physical activity or evaluate the effectiveness of any intervention; therefore, no specific activity format can be recommended on the basis of the current findings.

eHealth literacy may also be relevant to student support because university students frequently use electronic sources to obtain health information. Previous research among college students found that higher eHealth literacy was associated with greater engagement in health-promoting behaviors, including interpersonal support, exercise, and stress management (28). Longitudinal evidence from Chinese college students further showed that baseline eHealth literacy was prospectively associated with health-promoting lifestyles and health-related quality of life at follow-up, although these findings did not establish causality (29). Moreover, a study involving students from three Chinese universities found positive associations among eHealth literacy, physical literacy, and physical activity, with moderate-to-vigorous physical activity partially mediating the association between eHealth literacy and physical literacy (30). A study of young electronic media users in China also found that higher mental health-related eHealth literacy was associated with more positive attitudes toward seeking mental health support and better mental wellbeing (31). These findings suggest that educational initiatives aimed at strengthening students' ability to locate, evaluate, and apply online health information may be worth exploring. However, whether improving eHealth literacy contributes to better social adaptability remains to be examined in longitudinal or intervention studies.

Taken together, integrated student support strategies that include opportunities for physical activity and eHealth literacy education may warrant further evaluation. These implications should be regarded as hypothesis-generating directions rather than specific intervention recommendations.

### 4.4 Limitations

This study has several limitations that should be acknowledged. First, the sample was limited to first- and second-year students from a single medical college, which may limit the generalizability of the findings to other institutions, academic disciplines, and student populations. Second, participants were recruited through an online convenience sampling approach with voluntary participation, and the exact number of eligible students who received the survey invitation was not recorded. Therefore, a conventional response rate could not be calculated, and potential self-selection bias and limited representativeness cannot be excluded. Third, given the cross-sectional design, the study does not permit conclusions regarding causality or temporal ordering, and the observed relationships should be interpreted as statistical associations rather than causal effects. Moreover, the two survey waves involved separate cross-sectional samples rather than longitudinal follow-up of the same students. Although survey wave was included as a covariate in the regression analysis, residual cohort effects and differences in academic experiences between first- and second-year students cannot be fully excluded. Accordingly, the observed year-of-study difference should not be interpreted as evidence that social adaptability changes or declines over time. Fourth, all variables were assessed using self-report measures, which may be subject to recall, reporting, and social desirability biases. Fifth, pandemic-related experiences were not directly assessed, and no pre-pandemic comparison group was available. Therefore, the extent to which the findings were related to the broader post-pandemic educational context cannot be determined. Finally, the final regression model had modest explanatory capacity, with an adjusted *R*^2^ of 16.6%, indicating that a substantial proportion of the variability in social adaptability remained unexplained. Several potentially relevant individuals, family, interpersonal, and educational factors, including baseline mental health, family background, academic stress, and perceived social support, were not assessed. Future studies should use larger, multicenter samples and longitudinal designs, incorporate a broader range of contextual and psychological variables, and, where feasible, include objective or multi-informant measures to further examine the robustness and temporal nature of these associations.

## 5 Conclusion

This study examined the associations of eHealth literacy and physical activity with social adaptability among first- and second-year medical students at Wannan Medical College. Social adaptability was positively associated with both eHealth literacy and physical activity, and these associations remained statistically significant after adjustment for selected sociodemographic characteristics and survey wave. Gender, year of study, place of origin, program duration, and student leadership status were also associated with social adaptability.

Given the cross-sectional design, causal inferences cannot be drawn. Nevertheless, the findings suggest that eHealth literacy and physical activity may represent two relevant cognitive and behavioral factors associated with social adaptability among early-year medical students. These results provide a basis for future longitudinal and intervention studies and may inform the development of student support initiatives aimed at promoting psychosocial adjustment during the transition to university life.

## Data Availability

The raw data supporting the conclusions of this article will be made available by the authors, without undue reservation.
